# Peptide Human Neutrophil Elastase Inhibitors from Natural Sources: An Overview

**DOI:** 10.3390/ijms23062924

**Published:** 2022-03-08

**Authors:** Lorenza Marinaccio, Azzurra Stefanucci, Giuseppe Scioli, Alice Della Valle, Gokhan Zengin, Angelo Cichelli, Adriano Mollica

**Affiliations:** 1 Department of Pharmacy, “G. d’Annunzio” University of Chieti-Pescara, 66100 Chieti, Italy; lorenza.marinaccio@unich.it (L.M.); giuseppe.scioli@unich.it (G.S.); alice.dellavalle@unich.it (A.D.V.); a.mollica@unich.it (A.M.); 2 Department of Biology, Science Faculty, Selcuk University, Konya 42130, Turkey; gokhanzengin@selcuk.edu.tr; 3 Department of Medical, Oral and Biotechnological Sciences, “G. d’Annunzio” University Chieti-Pescara, Via dei Vestini 31, 66100 Chieti, Italy; angelo.cichelli@unich.it

**Keywords:** elastase inhibitors, human neutrophil elastase, pancreatic elastase, natural peptide, depsipeptide

## Abstract

Elastases are a broad group of enzymes involved in the lysis of elastin, the main component of elastic fibres. They are produced and released in the human body, mainly by neutrophils and the pancreas. The imbalance between elastase activity and its endogenous inhibitors can cause different illnesses due to their excessive activity. The main aim of this review is to provide an overview of the latest advancements on the identification, structures and mechanisms of action of peptide human neutrophil elastase inhibitors isolated from natural sources, such as plants, animals, fungi, bacteria and sponges. The discovery of new elastase inhibitors could have a great impact on the pharmaceutical development of novel drugs through the optimization of the natural *lead compounds*. Bacteria produce mainly cyclic peptides, while animals provide for long and linear amino acid sequences. Despite their diverse natural sources, these elastase inhibitors show remarkable IC_50_ values in a range from nM to μM values, thus representing an interesting starting point for the further development of potent bioactive compounds on human elastase enzymes.

## 1. Introduction

Elastic fibres compose an essential part of the extracellular matrix of connective tissue, where they allow its reversible and repetitive deformation. Each elastic fibre is mainly constituted by an amorphous core of elastin, which occupies more than 90% of the fibre, surrounded by an external envelope of microfibrils [1,2]. Fibrillin microfibrils ensure extensibility and elasticity during the deformation of tissues. A huge variety of fibrillin-binding proteins occurs for the assembly of elastic fibres [3], which play an important role in ensuring mechanical resilience, durability and cell interactivity within tissues [4].

Elastin is a hydrophobic protein that displays a huge cross-linking among Lys residues following the oxidation performed by the lysil-oxidase enzyme, which gives elastin a high level insolubility [1]. This protein contains a large quantity of hydrophobic amino acids. In particular, higher vertebrates’ elastin is composed of more than 30% Gly and about 75% Val, Ala and Pro residues [1,5]. The formation process of elastic fibres is called *elastogenesis,* comprising tropoelastin (elastin’s subunits) synthesis, coacervation, cross-linking and deposition [4].

Elastases are a group of enzymes that selectively lyse the amorphous component of elastic fibre, namely elastin [2]. Elastases are produced and released in different locations of the human body. Among them, human neutrophil elastase and pancreatic elastase show an important physiological and pathological role. In detail, pancreatic elastase measurement in faeces (faecal elastase-1) is used as an indirect test to determine the onset of the exocrine pancreatic insufficiency (EPI). Pancreatic elastase is secreted from pancreatic acinar cells and after binding to the bile salts goes through a tiny degradation into the gut. Clinicians can make use of two possible ELISA tests to measure faecal elastase-1 through monoclonal or polyclonal antibodies [6]. For example, the ScheBo Pancreatic Elastase 1 Stool Test (ScheBo Biotech, Giessen, Germany) is one of the most-used tests based on two monoclonal antibodies that measure the isoforms chymotrypsin-like elastase 3B (CELA3B) and, with lower efficiency, CELA3A [7].

**Figure 1:**
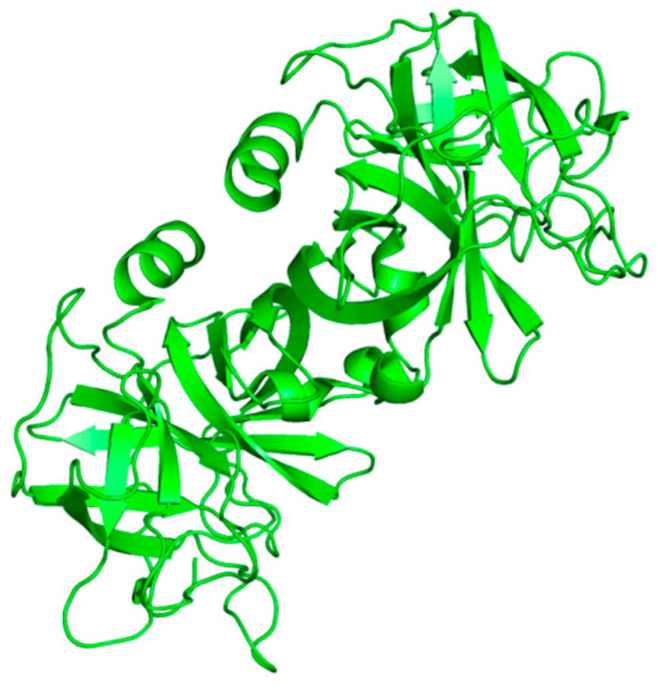
Structure of free human neutrophil elastase (HNE) from the RCSB Protein Data Bank (PDB-ID: 3Q76) [15,16].

**Scheme 1:**
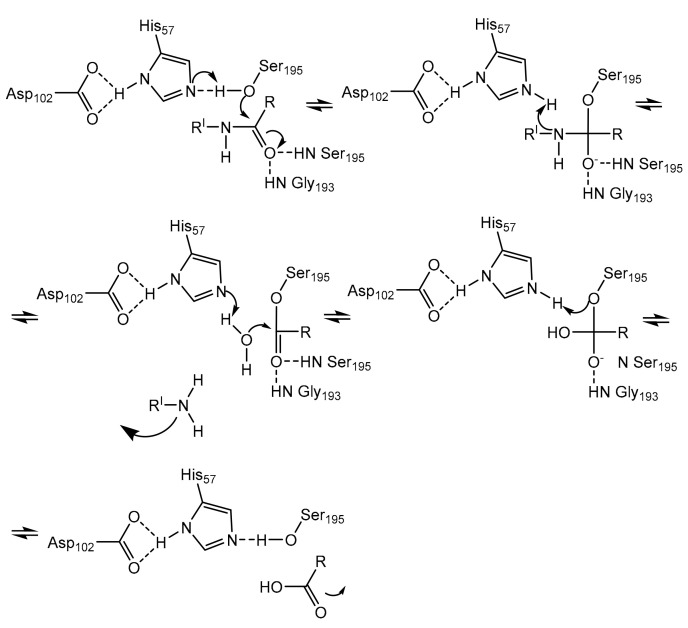
Mechanism of action of serine proteases [11].

Neutrophils, a large population of leukocytes, play an important role in the regulation of the innate and adaptive immune responses, quickly achieving the inflammation/infection site [8]. During the degranulation process, neutrophils release human neutrophil elastase (HNE) (Figure 1), a glycoprotein composed of 218 amino acids, through extracellular neutrophil traps (NETs) [8,9]. NETs are composed of decondensed chromatin, neutrophil elastase and other enzymes [8]. HNE is a serine protease belonging to the chymotripsin family that is produced in the bone marrow during the stage of promyelocytes [9]. Neutrophil serine proteases are involved in inflammation and the immune response [10]. HNE is deposited as an active enzyme in the azurophilic granules of polymorphonuclear neutrophils. This enzyme has a catalytic site of three amino acids, called the catalytic triade: Asp102, His57 and Ser195. The mechanism of action of serine proteases, which includes neutrophil elastase, is illustrated in Scheme 1; when the target ligand achieves the enzymatic catalytic site, a proton transfer happens among the three amino acids, causing an increased nucleophile activity of Ser195 with a concomitant peptide bond cleavage [9,11]. In regular conditions, a balance between human neutrophil elastase activity and its endogenous inhibitors (e.g., elafin, serpins, α1-antitrypsin and secretory leukocyte proteinase inhibitor) is guaranteed, while the imbalance between elastase activity and its endogenous inhibitors can cause a variety of illnesses, such as chronic obstructive pulmonary disease, acute lung injury, acute respiratory distress syndrome and pulmonary fibrosis [12,13]. It has recently been hypothesized that neutrophil elastase inhibitors could be used to treat acute respiratory distress syndrome (ARDS) caused by COVID-19 infection, but their effective use is yet unproven, and more clinical studies should be conducted to evaluate the response and effectiveness of these types of inhibitors in COVID-19 patients [14].

The discovery of new elastase inhibitors from natural sources, such as plants, animals, fungi, bacteria and sponges, could have a great impact on the pharmaceutical development of novel drugs to prevent and to treat diseases caused by the excessive activity of elastase or the pathological degradation of elastin. Thus, the main aim of this review is to expose the latest advancements in the identification of peptide inhibitors of human elastase isolated from natural sources, trying for the first time, to the best of our knowledge, to outline all the natural peptide compounds active on human neutrophil elastase (also called human leukocyte elastase). We will provide an overview of their origins, structures and mechanisms of action where already known. This review could be the starting point for further investigations on the structural features necessary to promote a better interaction among peptide compounds and the active site of human neutrophil elastase, leading to the future synthesis of more potent peptide inhibitors. The development of novel peptide HNE inhibitors could be beneficial for the treatment of chronic obstructive pulmonary disease, acute lung injury, pulmonary fibrosis and disorders caused by COVID-19 infection, such as acute respiratory distress syndrome.

## 2. Natural Peptide Elastase Inhibitors Isolated from Animal Sources

### 2.1. ShSPI from Scolopendra Hainanum

**Figure 2:**
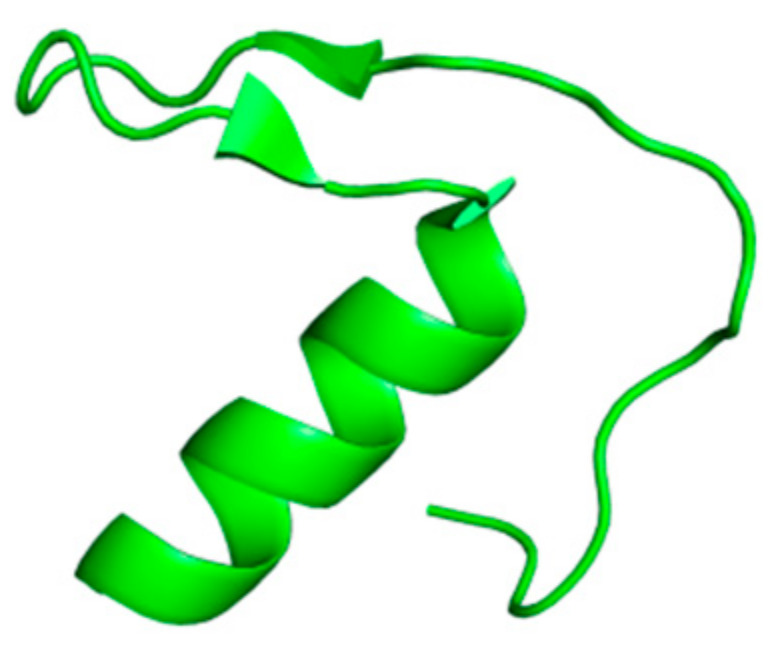
The solution structure of ShSPI (NMR ensemble) from the RCSB Protein Data Bank (PDB-ID: 6LF5) [12,16].

Luan et al. discovered a new serine protease inhibitor from the cDNA library of the venom glands of centipede *Scolopendra Hainanum*. This compound is called ShSPI, and it has been identified as an atypical kazal-type protease inhibitor that could be developed as a novel drug for the treatment of illnesses caused by human elastase. ShSPI is a bioactive peptide composed of 34 amino acids whose primary sequence is: CPQVCPAIYQPVFDEFGRMYSNSCEMQRARCLRG. The three-dimensional structure (Figure 2) displays a cystine-stabilized α-helix formed by Ser23 and Arg33 and a two-stranded anti-parallel β-sheet (Pro11 to Asp14 and Gly17 to Tyr20); the folding is guaranteed by the tertiary structure and the presence of two disulfide bonds among Cys1-Cys31 and Cys5-Cys24 [12]. Its inhibitory activity was compared to Sivelestat (ONO-5046), a human neutrophil elastase inhibitor tested as clinical drug for the treatment of ARDS and recently taken in consideration for the management of acute lung injury/acute respiratory distress syndrome (ALI/ARDS) or ARDS with coagulopathy caused by COVID-19 infection [12,17,18]. Both compounds inhibit HNE in a dose-dependent manner, but ShSPI exhibits a more effective activity than Sivelestat at the same concentration. ShSPI acts with a non-competitive inhibition mechanism against elastase, displaying a K_i_ value of 12.6 nM, and its equilibrium dissociation constant (K_D_) to HNE is 4.2 × 10^−8^; furthermore, ShSPI shows a huge stability after co-incubation with human plasma over 48 h [12].

### 2.2. AvKTI from Araneus Ventricosus

Peptide AvKTI has been isolated from a spider (*Araneus ventricosus*); it has the ability to inhibit trypsin, chymotrypsin, plasmin and human neutrophil elastase. It is composed of 170 amino acids and comprises a signal peptide of 19 units, a pro-peptide of 94 building blocks and a mature peptide of 57 amino acids that represents a Kunitz domain. The mature AvKTI has a peptide sequence (KDRCLLPKVTGPCKASLTRYYYDKDTKACVEFIYGGCRGNRNNFKQKDECEKACTDH) similar to other Kunitz-type serine protease inhibitors. This compound is the first Kunitz-type serine protease inhibitor isolated from spiders to be known for its antifibrinolytic and antielastolytic activity [19]. Kunitz-type inhibitors are usually composed of a single inhibitory domain or a multi-domain included in a single-chain compound [20]. It is produced in the spider’s epidermis; however, the physiological role of this compound in spiders is not yet clear [19,20]. AvKTI inhibits neutrophil elastase with an IC_50_ value of 446.93 nM and plasmin with an IC_50_ value of 10.07 nM, showing a lower neutrophil elastase inhibitory activity of about 44.4-fold. The K_i_ value of AvKTI against neutrophil elastase is 169.07 nM, while its K_i_ value against plasmin is 4.89 nM [19].

### 2.3. Gaumerin from Hirudo Nipponia

Guamerin is a cysteine-rich polypeptide composed of 57 amino acids (VDENAEDTHGLCGEKTCSPAQVCLNNECACTAIRCMIFCPNGFKVDENGCEYPCTCA) with a molecular weight of 6.110 that was isolated from a native Korean leech *Hirudo nipponia* by Jung et al. Its structure is characterized by ten cysteine residues that confer a high rigidity due to the formation of disulfide bridges. Guamerin showed stability at 25 °C in a range of pH varying from 1 to 11. It has the ability to inhibit human leukocyte elastase (HLE) with a K_i_ value of 8.1 × 10^−14^ M [21].

## 3. Natural Peptide Elastase Inhibitors Isolated from Fungi

### 3.1. Desmethylisaridin C2, Isaridin E, Isaridin C2 and Roseocardin from Beauveria Felina

**Figure 3:**
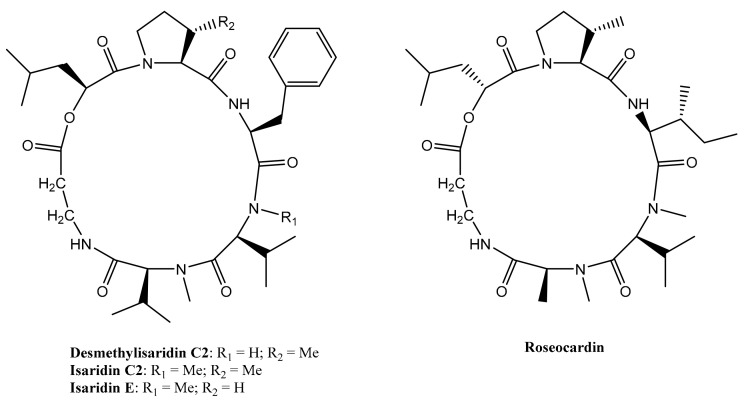
Structures of Desmethylisaridin C2, isaridin E, isaridin C2 and roseocardin.

Eight cyclodepsipeptides have been produced by the filamentous fungi *Beauveria felina* after the addition of suberoylanilide hydroxamic acid (SAHA), a histone deacetylase (HDAC) inhibitor, to the culture medium; three of them are new compounds, e.g., desmethylisaridin C2, desmethylisaridin E and isaridin F [22]. This is an example of an epigenetic tool used for obtaining inhibitor peptides from fungal sources [23]. Among them, Desmethylisaridin C2, isaridin E, isaridin C2 and roseocardin (Figure 3) are able to inhibit elastase release induced by formyl-L-methionyl-L-leucyl-L-phenylalanine (FMLP) in human neutrophils and to express an anti-inflammatory action without toxicity for human neutrophils. The IC_50_ values of these compounds are displayed in Table 1 [22].

### 3.2. AFUEI from Aspergillus fumigatus

AFUEI is an elastase inhibitor purified by Okumura et al. from *Aspergillus fumigatus* strain AFU-12 that came from the sputum of a patient afflicted with allergic bronchopulmonary aspergillosis. Its primary amino acid structure is composed of 68 residues (DPATCEKEAQFVKQELIGQPYTDAVANALQSNPIRVLHPGDMITMEYIASRLNIQVNENNEIISAHCA), and the molecular mass of its protein portion is 7526.2 Da. AFUEI could be effective against inflammatory diseases since it is able to inhibit human leukocyte elastase with an inhibitory activity of 83.8% [24]. *Aspergillus fumigatus* synthesizes *A. fumigatus* elastase (AFUE) and native *A. fumigatus* elastase inhibitor (N-AFUEI) in liquid medium, but N-AFUEI is barely produced. Therefore, for medical purposes, synthetic-AFUEI (S-AFUEI) has been produced by Peptide Institute Inc. (Osaka, Japan) since 2015. Further investigations about S-AFUEI’s biological and physiological activities have been reported by Fukui et al. [25].

## 4. Cyclic Peptides Derived from Bacteria as Elastase Inhibitors

### 4.1. Lyngbyastatin 4 and Lyngbyastatin 7

**Figure 4:**
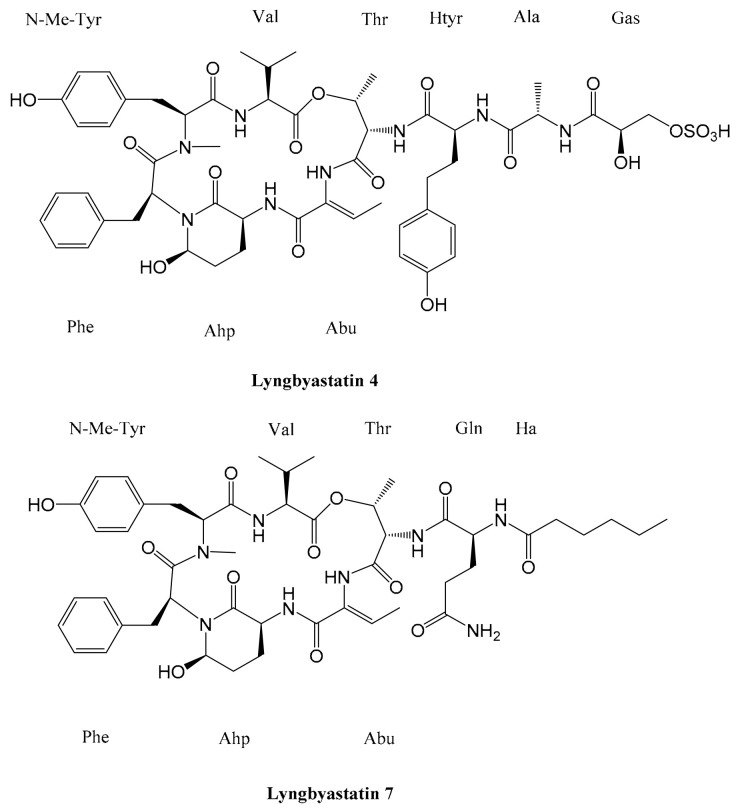
Structures of Lyngbyastatin 4 and Lyngbyastatin 7.

Cyanobacteria are ancient forms of life highly spread on our planet and seem to be very interesting in order to promote pharmaceutical advancements due to their secondary metabolites with a typical restricted conformation and their related metabolic stability and biological activities [26,27]. Cyclic depsipeptides isolated from cyanobacteria show similarities in their structures, which are usually composed of an amino acid sequence of six residues assembling a macrocyclic ring. The majority of these compounds display a residue of 3-amino-6-hydroxy-2-piperidone (Ahp). More than 20 depsipeptides show a hexadepsipeptide core, a 2-amino-2-butenoic acid (Abu) residue next to the Ahp ring and variable side chains (Figure 4). Another similar feature of these depsipeptides is the ester linkage between the hydroxyl group of L-threonine located in the β position and the carboxyl group of the last amino acid in *C*-terminal position [28].

Different peptides are produced by marine cyanobacteria belonging to genus *Lyngbya* [23]. *Lyngbya confervoides* coming from the Florida Atlantic coast is a producer of lyngbyastatin 4, a depsipeptide showing two peculiar residues of homotyrosine and Ahp; it has the ability to inhibit human neutrophil elastase, with an IC_50_ value of 49 nM. Moreover, it is not considered cytotoxic to several cancerous cell lines [29,30].

Lyngbyastatin 7 is a potent compound isolated from *Lyngbya* spp. whose structure has been defined by Taori et al. It has been tested by Salvador et al. as an inhibitor at a single concentration against 68 proteases. It exhibits a specific activity against serine protease elastase, proteinase K and chymotrypsin; it is able to inhibit HNE, with an IC_50_ value of 23 nM [29,31]. Its structure is characterized by a cyclic depsipeptide core composed of 19 residues, including the 3-amino-6-hydroxy-2-piperidone (Ahp) [9,31]. A low quantity of Lyngbyastatin 7 can be isolated from the natural source. Therefore, total synthesis methodologies have been developed to investigate its activity [32,33]. Salvador et al. elucidated the interaction between lyngbyastatin 7 and porcine pancreatic elastase through co-crystallization using the hanging drop vapor diffusion technique with the aim to better understand the mechanism of inhibition; Lyngbyastatin 7 and its related compounds act as substrate mimics. Indeed, the subsites S1 to S4 of the enzyme are occupied by the 2-amino-2-butenoic acid (Abu) and the *N*-terminal amino acids [29].

### 4.2. Tutuilamides A–C

**Figure 5:**
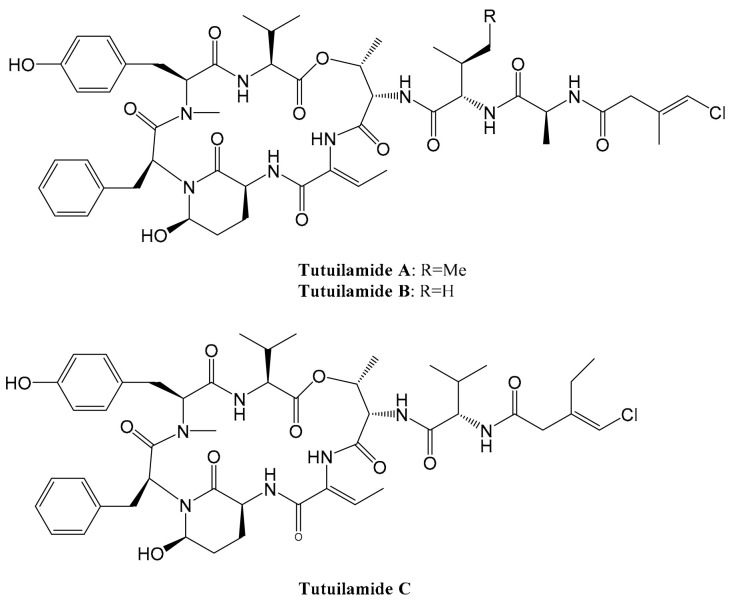
Structures of Tutuilamides A–C.

Keller et al. isolated three new peptides containing Ahp and Abu residues from two different bacteria, tutuilamide A and tutuilamide B from cyanobacterium *Schizothrix* sp. and tutuilamide C from *Coleofasciculus* sp. Their structures are characterized by an uncommon vinyl-chloride-containing residue. Tutuilamides A and B show the same cyclic core, but they differ for an amino acid (isoleucine in place of valine). The primary sequence of Tutuilamide A is Val-*N*-MeTyr-Phe-Ahp-Abu-Thr-Ile-Ala-Cmb. Tutuilamide C is similar to Tutuilamide B, except for the lack of an alanine residue and the presence of an additional methylene in the side chain (Figure 5). Tutuilamides A–C have been tested for the inhibition activity against serine proteases, e.g., porcine pancreatic elastase (PPE). Tutuilamides A and B are the most active as inhibitors against PPE, with an IC_50_ of 1-2 nM [28]. Keller et al. also investigated the crystallized structure of the complex between Tutuilamide A and porcine pancreatic elastase, revealing a good interaction in the substrate binding pocket and the presence of a supplemental H-bond formed between the carbonyl group of the Cmb residue and the backbone amide of Arg217 not observed in the lyngbyastatin 7 co-crystalized structure [28,29]. Therefore, Tutuilamide A shows a higher potency than lyngbyastatin 7 against PPE [28].

The total synthesis of Tutuilamide A has been reported through late-stage diversification, a convergent synthetic strategy similar to the total synthesis of lyngbyastatin 7 [32,33,34]. Its inhibitory activity against HNE has been evaluated, showing a high selectivity for this enzyme [34]. Tutuilamide A shows a higher inhibitory activity against PPE than lyngbyastatin 7. On the contrary, lyngbyastatin 7 seems to be equal or better than it in inhibiting HNE [28,34]. The IC_50_ values of Tutuilamide A and lyngbyastatin 7 against HNE are 0.73 nM and 0.85 nM, respectively [34].

### 4.3. Loggerpeptins A–C and Molassamide

**Figure 6:**
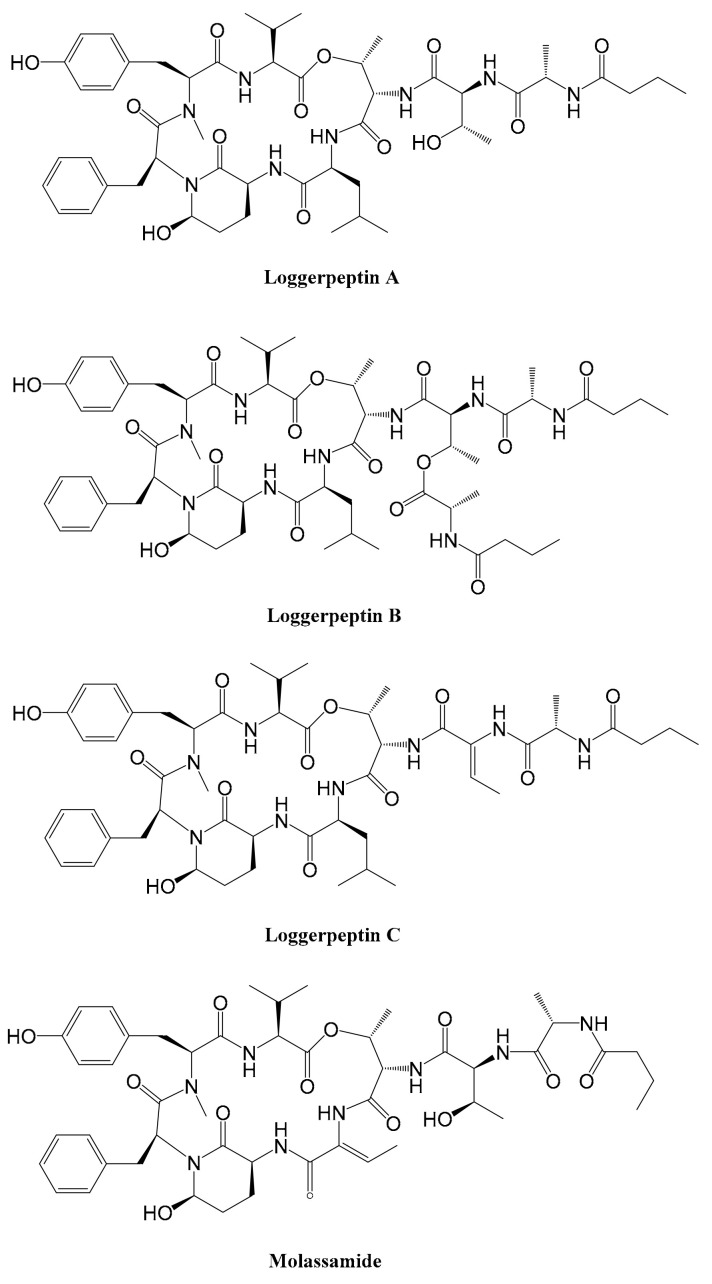
Structures of Loggerpeptins A–C and Molassamide.

Al-Awadhi et al. identified four serine protease inhibitors from the marine cyanobacterium DRTO-73 of Loggerhead Key in Florida: Loggerpeptins A–C and molassamide. Loggerpeptins A–C have different molecular formulas: C_50_H_72_N_8_O_13_, C_57_H_83_N_9_O_15_ and C_50_H_70_N_8_O_12_, respectively [35]. Molassamide (C_48_H_66_N_8_O_13_) is a compound previously isolated from the cyanobacterium *Dichothrix utahensis* by Gunasekera et al. who elucidated its structure and inhibitory activity against porcine pancreatic elastase, α-chymotrypsin from bovine pancreas and trypsin from porcine pancreas (Figure 6) [36]. Loggerpeptins A–C and molassamide display a similar core structure composed of a 19-membered cyclic hexadepsipetide, including the Ahp residue and a modified glutamic acid residue. All the compounds have been tested against human neutrophil elastase and compared with sivelestat inhibition (IC_50_ = 0.06 μM), showing the ability to inhibit HNE with IC_50_ values of 0.29 μM, 0.89 μM, 0.62 μM and 0.11 μM, respectively. Among them, the most potent inhibitor is molassamide, while loggerpeptins B and C show less activity than loggerpeptin A. Loggerpeptin C differs from its analogues because of the presence of an Abu unit within the side chain [35].

### 4.4. Symplostatins 5–10

**Figure 7:**
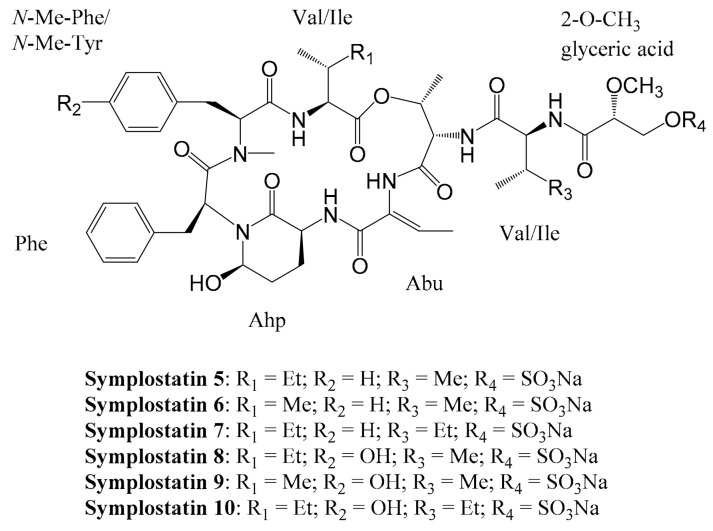
Structures of Symplostatins 5–10.

Symplostatins 5–10 are six cyclic depsipeptides containing an Ahp moiety derived from the red marine cyanobacterium *Symploca* sp. While symplostatins 8–10 contain an *N*-Me-Tyr residue, symplostatins 5–7 contain an *N*-Me-Phe residue (Figure 7). Salvador et al. evaluated their antiproteolytic activity against human neutrophil elastase. Among them, symplostatins 8–10 exhibit a more potent and effective inhibition of HNE (IC_50_ values of 41 nM, 28 nM and 21 nM, respectively). These three compounds, along with lyngbyastatins 4 and 7, are more effective in elastase inhibition than sivelestat, a specific human neutrophil elastase inhibitor. Further investigations on the inhibitory activity of symplostatins 5–10 towards human and bovine pancreatic chymotrypsin have been conducted, showing less potency compared to elastase inhibition [29].

### 4.5. Insulapeptolides A–H

**Figure 8:**
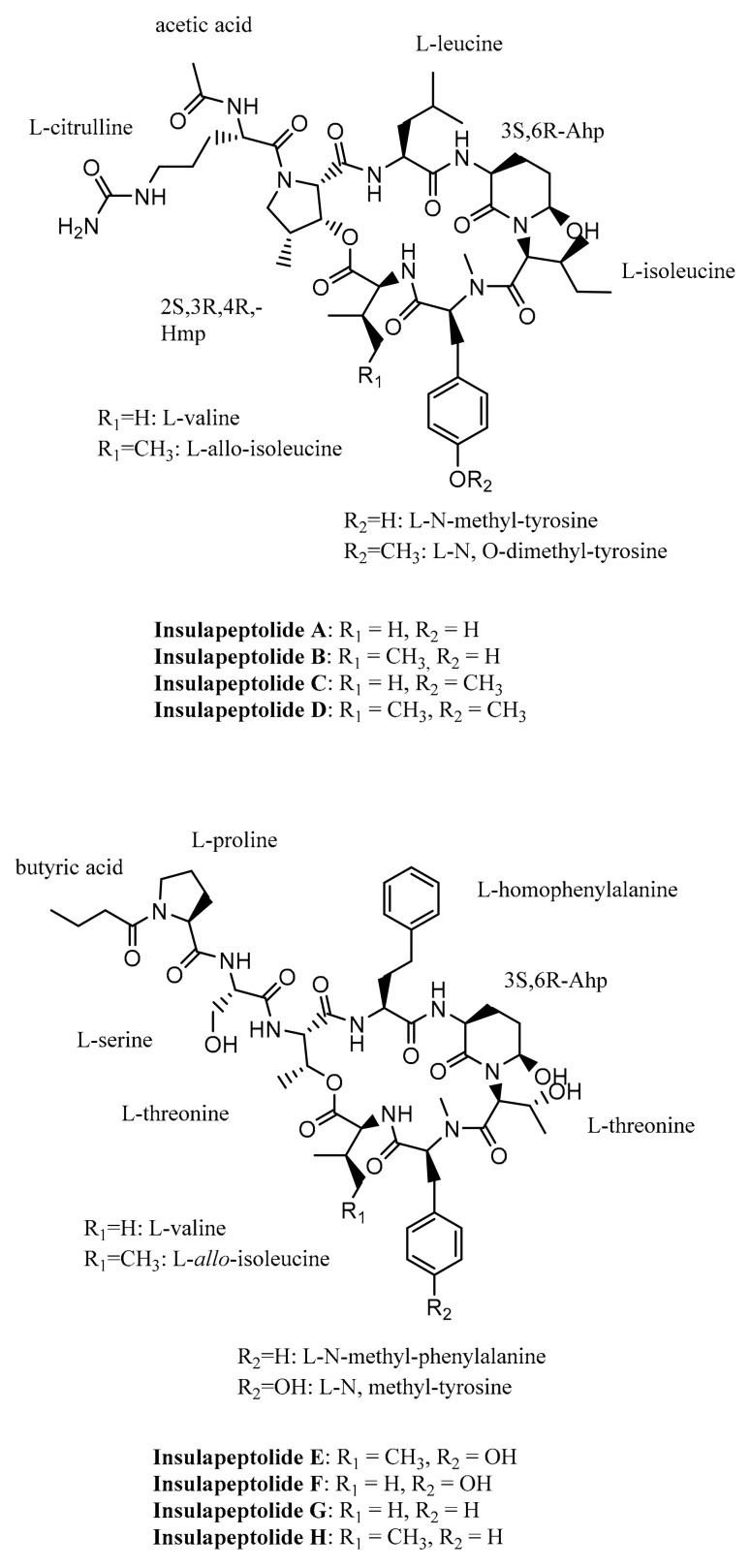
Structures of Insulapeptolides A–H.

Following the evaluation of 17 cyanobacterial strains of various genera in a human leukocyte elastase inhibition assay, Mehner et al. identified the strain *Nostoc insulare* as an important source of HLE inhibitors. They isolated eight cyanopeptolins, namely insulapeptolides A–H, which show the ability to inhibit HLE, with IC_50_ values in a range from micro- to nanomolar values (Table 2). Insulapeptolides A–D exhibit two peculiar amino acids, 3-hydroxy-4-methyl-proline (Hmp) and citrulline, while insulapeptolides E–H display the L-threonine residue rather than Hmp moiety; these compounds show less inhibitory activity towards HLE (Figure 8). Moreover, Mehner et al. suggest that insulapeptolides act on HLE through a competitive inhibitory mechanism [26].

### 4.6. Brunsvicamides A–C

**Figure 9:**
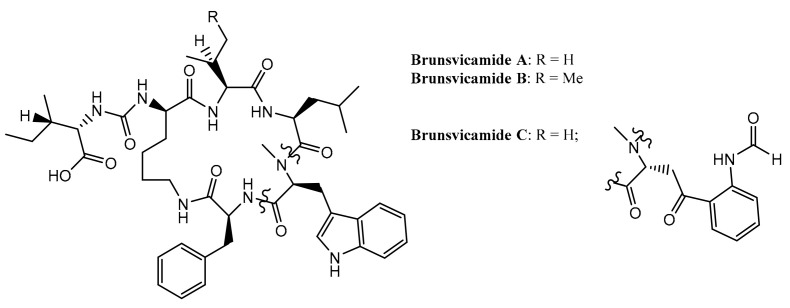
Structures of Brunsvicamides A–C.

Brunsvicamides A–C, isolated from *Tychonema* sp., are cyclic peptides composed of six amino acids: five of them are enclosed in the 19-membered ring skeleton and the last one is linked to the α-amino group of the D-Lys thanks to the urea moiety (Figure 9). The cyclic ring is locked through an amide bond between Phe and D-Lys residues. Brunsvicamides A and B are characterized by the presence of L-amino acids, while brunsvicamide C shows an *N*-methyl-*N*^I^-formylkynurenine derived from tryptophan in the D-configuration [27,37]. These compounds show a selective activity against human leukocyte elastase, with K_i_ values of 1.1 μM, 0.70 μM and 1.6 μM, respectively, measured considering a competitive inhibition mechanism, and IC_50_ values of 3.12 μM, 2.00 μM and 4.42 μM, respectively [27].

### 4.7. FR901277

**Figure 10:**
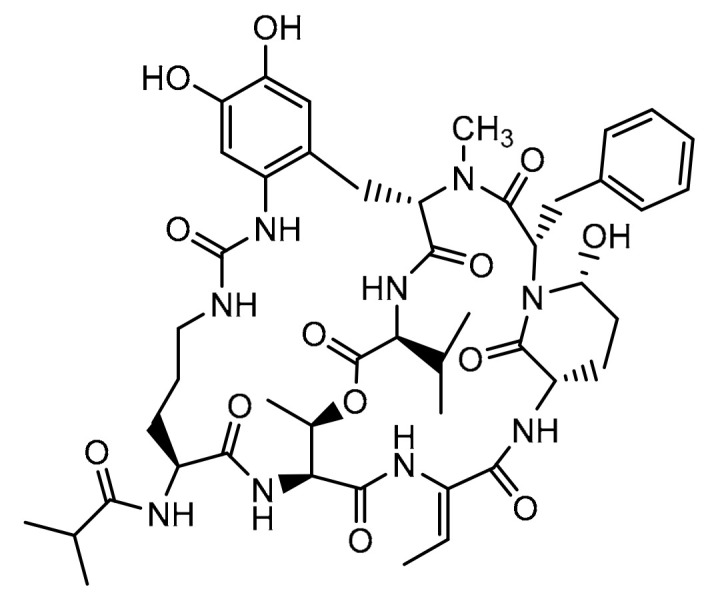
Structure of FR901277.

FR901277 is a natural compound identified and isolated from the bacterium filtrate of *Streptomyces resistomycificus* that acts as an HLE inhibitor (IC_50_ value of 1.8 × 10^−7^ M) (Figure 10). It displays a competitive inhibition mechanism against elastase (K_i_ = 1.2 × 10^−8^ M) [38]. It has a bicyclic structure with an isopropyl carbonyl *N*-terminus, four common amino acids and three unusual ones [39].

### 4.8. YM-47141 and YM-47142

**Figure 11:**
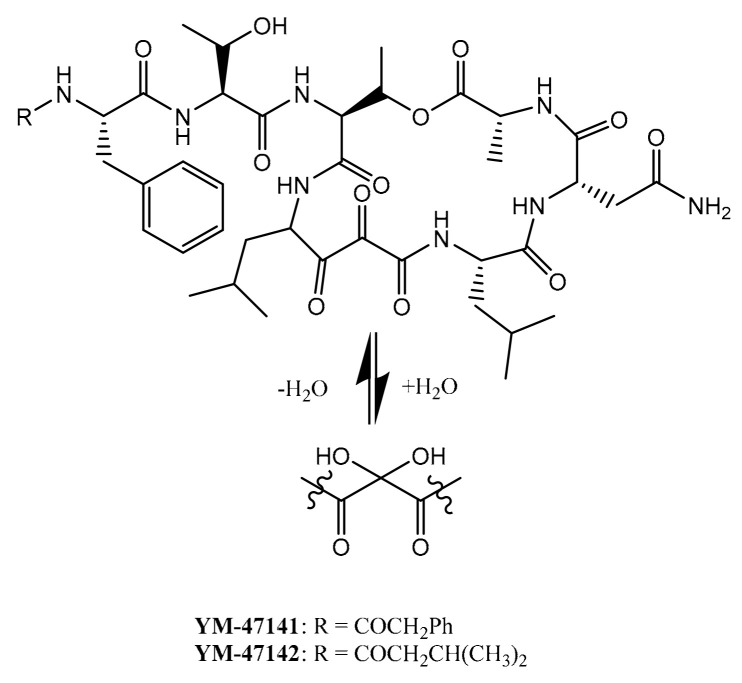
Structures of YM-47141 and YM-47142.

YM-47141 (C_46_H_62_N_8_O_13_) and YM-47142 (C_43_H_64_N_8_O_13_) are cyclic depsipeptides that were discovered by Yasumuro et al. from the fermented bacterium *Flexibacter* sp. strain Q17897 obtained from a soil sample and belonging to Gram-negative bacteria (Figure 11). These compounds are human leukocyte elastase inhibitors with IC_50_ values of 1.5 × 10^−7^ M and 3.0 × 10^−7^ M, respectively; among them, YM-47141 exhibits a superior inhibitory activity against HLE. Their structures are particularly interesting because of a vicinal tricarbonyl function; these are the first protease inhibitors containing this function discovered from a microbial source. They suggest that the elastase inhibitory activity is due to the formation of hemiketal between the serine residue on the catalytic enzymatic site and the 2-carbonyl group of 2,3-dioxo-4-amino-6-methyl-heptanoic acid contained in YM-47141 and YM-47142 [40].

## 5. Peptide Elastase Inhibitors Isolated from Plants

### 5.1. Ixorapeptide I and II from Ixora Coccinea

**Figure 12:**
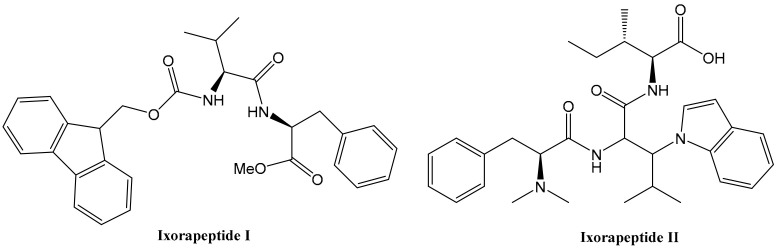
Structures of Ixorapeptide I and Ixorapeptide II.

Ixorapeptide I and II are derivatized peptides isolated from the methanol extract of the aerial part of *Ixora coccinea* by Lee et al. (Figure 12). Only Ixorapeptide II has the ability to inhibit the elastase release in the anti-inflammatory assay (IC_50_ value of 0.27 μg/mL), showing a possible anti-inflammatory activity on neutrophils. It is 73 times more active on elastase release inhibition than phenylmethylsulfonyl fluoride (PMSF), a commercial reference compound, without the relevant cytotoxicity in the cancer cell lines [41].

### 5.2. Roseltide rT1 from Hibiscus sabdariffa

**Figure 13:**
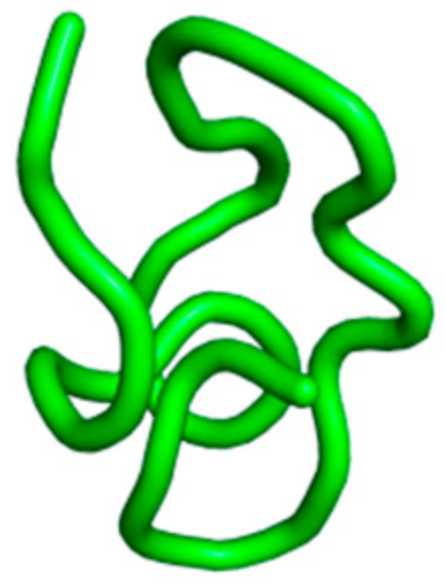
Structure of Roseltide rT1 (NMR ensemble) from the RCSB Protein Data Bank (PDB-ID: 5GSF) [16,42].

Roseltide rT1 (Figure 13) has been isolated from a medicinal plant called *Hibiscus sabdariffa* that belongs to the Malvaceae family. Loo et al. conducted a proteomic and transcriptomic analysis revealing eight cysteine-rich peptides called roseltides (rT1–rT8) containing six cysteine residues and derived from a three-domain precursor composed of 90 residues. The most abundant among them, Roseltide rT1, is composed of 27 residues, and it is defined as a knottin-type elastase inhibitor characterized by a cysteine-knot disulfide linkage with a cysteine spacing pattern of CX_6_CX_6_CCX_4_CX_4_CX. In total, 85% of its amino acids are hydrophobic. Overall, it is a positively charged peptide. It is able to inhibit human neutrophil elastase in a dose-dependent manner, showing an IC_50_ value of 0.47 μM. The presence of disulfide bridges in the structure of roseltide rT1 gives it an increased resistance against the acid degradation promoted by proteinases and human serum [42].

Moreover roseltide rT1 shows some features common to mitochondria-targeting compounds and helical peptides containing a huge quantity of Cys and Leu residues; it has been defined as a new class of mitochondria-targeting molecules different from the mitochondria-targeting helical peptides already known, showing a non-helical structure composed of four “clover-like” loops containing three disulfide bonds [43].

## 6. Cyclic Peptides from Marine Sponges

**Figure 14:**
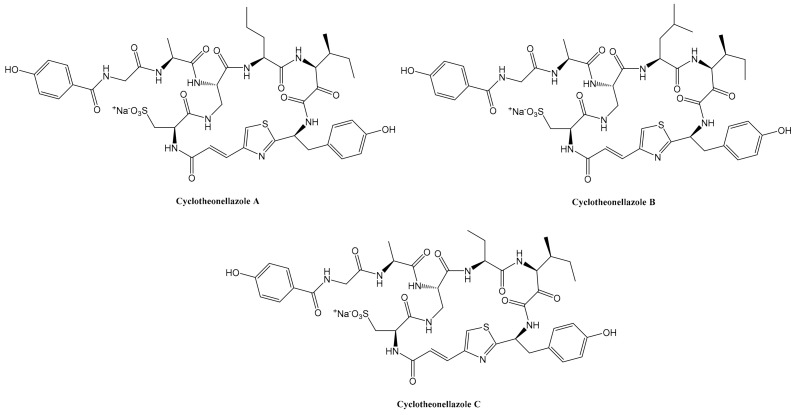
Structures of Cyclotheonellazoles A–C.

Peptides isolated from marine organisms often exhibit cyclic structures with substituted *N*- or *C*-termini. Therefore, they are relatively non-polar and easy to separate and purify from marine source. These compounds are often characterized by uncommon amino acids, including non-proteinogenic D-amino acids [44]. A huge number of bioactive secondary metabolites have been discovered from marine sponges of the genus *Theonella,* including peptides; Issac et al. isolated three novel cyclic peptides called cyclotheonellazoles A–C, in the sponge *Theonella* aff. *Swinhoei* from Dos de la Baleine, Madagascar [45]. They belong to the azole-homologated peptides (AHPs) that are characterized by the presence of azole-homologated amino acids. This class of peptides is produced by non-ribosomal synthase/polyketide synthase (NRPS-PKS) and are classified in two different types (I or II), depending on the presence of conserved tetrads of amino acids into the macrocyclic ring [44]. Cyclotheonellazole A (C_44_H_54_N_9_Na_2_O_14_S_2_), cyclotheonellazole B (C_45_H_57_N_9_NaO_14_S_2_) and cyclotheonellazole C (C_43_H_52_N_9_Na_2_O_14_S_2_) have 22 degrees of unsaturation, and their structures include few proteinogenic amino acids (two or three) and non-proteinogenic acids, e.g., 4-propenoyl-2-tyrosylthiazole (Ptt), 3-amino-4-methyl-2-oxohexanoic acid (Amoha) and diaminopropionic acid (Dpr). The 2-aminopentanoic acid contained in cyclotheonellazole A is replaced with leucine in cyclotheonellazole B and homoalanine in cyclotheonellazole C (Figure 14). These three compounds have the ability to inhibit elastase with IC_50_ values of 0.034 nM, 0.10 nM and 0.099 nM, respectively. The most effective among them against elastase activity is cyclotheonellazole A, probably due to a better interaction with the enzyme S2 subsite [45].

Cyclotheonellazole A was isolated in low yield from the natural source, so Cui et al. recently defined its chemical synthesis to further investigate its possible pharmaceutical development as *lead compound*. Moreover, they tested the synthesized Cyclotheonellazole A against human neutrophil elastase, showing an IC_50_ value of 0.321 µM. Therefore, they proved that this compound is a more effective inhibitor against elastase than sivelestat (IC_50_ = 0.704 µM), showing an inconsistent result compared with the previous study conducted by Issac et al.; this is probably due to different experimental conditions, such as the source and concentration of the enzyme, the substrate and the time of incubation [45,46]. Elastase has recently been considered as a potential target for the prevention of ARDS in patients afflicted with SARS2-CoV-2 infection [14]. The high elastase inhibitory activity of this compound could be particularly advantageous for patients with a D614G genotype infection [46].

## 7. Conclusions

A huge number of bioactive molecules have been isolated from natural sources [47,48]. Among them, distinctive peptides have been discovered so far from plants, fungi, animals, sponges and bacteria. Particularly important are peptide human elastase inhibitors that inhibit an enzyme able to lyse the main component (˃90%) of elastic fibres, called elastin [1,2]. The imbalance between human neutrophil elastase activity and its endogenous inhibitors can cause a broad range of diseases, such as chronic obstructive pulmonary disease, acute lung injury, acute respiratory distress syndrome and pulmonary fibrosis [12,13]. This is the main motivation of researchers to discover and isolate new and potent elastase inhibitors from natural sources. Furthermore, elastase has been recently considered as a potential target for the prevention of acute respiratory distress syndrome (ARDS) in COVID-19 patients and the possible use of neutrophil elastase inhibitors to prevent and to treat these disorders has been hypothesized. However, future clinical studies should be conducted to evaluate the effectiveness of elastase inhibitors on COVID-19 patients [14].

Peptide elastase inhibitors isolated from animal sources and fungi are often characterized by long and linear amino acid sequences [12,19,21,24]. Plants provide different types of peptide elastase inhibitors, such as the short peptide ixorapeptide II isolated from the MeOH extract of the aerial part of *Ixora coccinea* and the peptide roseltide rT1 from *Hibiscus sabdariffa* [41,42]. Bacterial sources provide cyclic peptides with peculiar residues that act as elastase inhibitors [26,28,29,30,31,35,36,37,38,40]. Marine sponges of the genus *Theonella* produce cyclotheonellazoles A–C, cyclic peptides belonging to azole-homologated peptides (AHPs) [44,45]. Despite the structural differences among them, these natural peptide elastase inhibitors show remarkable IC_50_ values in a range from nM to μM.

The future development of peptide analogues as HNE inhibitors would be a very innovative approach to fight a huge variety of disorders using very cheap, safe and easily produced compounds. Furthermore, the cyclic structure of the natural *lead compounds* could be advantageous for the design of novel pharmaceutical drugs because of their large surface area that is connected to their affinity and selectivity for the target; moreover, the restrained structure gives them less flexibility, enhancing their binding properties. Cyclic peptides are usually easily produced, modified and characterized and are considered to have a low toxicity [49].

In conclusion, the latest advancements on the isolation and characterization of peptide inhibitors against human neutrophil elastase will be the starting point for the future development of novel inhibitor drugs through the optimization of natural *lead compounds* and drug design processes. We consider the recent discoveries as a step forward to understand the structural features necessary to express the inhibitory activity against human neutrophil elastase and provide new commercial drugs against chronic obstructive pulmonary disease, acute lung injury, acute respiratory distress syndrome, pulmonary fibrosis and ARDS linked to COVID-19 infection.

## Figures and Tables

**Table 1:** IC_50_ values of Desmethylisaridin C2, Isaridin E, Isaridin C2 and Roseocardin in vitro on formyl-L-methionyl-L-leucyl-L-phenylalanine (FMLP)-induced human neutrophils [22].

Compounds	IC_50_ (μM)
Desmethylisaridin C2	10.01 ± 0.46
Isaridin E	12.76 ± 1.00
Isaridin C2	12.12 ± 0.72
Roseocardin	15.09 ± 0.28

**Table 2:** IC_50_ values of Insulapeptolides A–H for the inhibition of human leukocyte elastase (HLE) [26].

Compounds	IC_50_ (μM)
Insulapeptolide A	0.14 ± 0.01
Insulapeptolide B	0.10 ± 0.01
Insulapeptolide C	0.090 ± 0.001
Insulapeptolide D	0.085 ± 0.004
Insulapeptolide E	3.2 ± 0.2
Insulapeptolide F	1.6 ± 0.1
Insulapeptolide G	3.5 ± 0.1
Insulapeptolide H	2.7 ± 0.1

## Data Availability

Not applicable.
